# Durability of washing machines under real life conditions: Definition and application of a testing procedure

**DOI:** 10.1016/j.jclepro.2020.121222

**Published:** 2020-07-10

**Authors:** Rainer Stamminger, Alexander Bues, Felice Alfieri, Mauro Cordella

**Affiliations:** a Universität Bonn, Institut für Landtechnik, Nussallee 5, 53115, Bonn, Germany; b European Commission, Joint Research Centre (JRC), Directorate B – Growth and Innovation, Circular Economy and Industrial Leadership Unit, Edificio EXPO – C/Inca Garcilaso 3, 41092, Seville, Spain

**Keywords:** Circular economy, Durability, Eco-design, Testing, Washing machines

## Abstract

One of the global challenges of our economy is to meet the demand of a growing population while reducing resource consumption and waste production. This means moving from a linear to a more circular economy model, in which the design of more durable products plays a key role. From a technical perspective, product design needs to be supported by sound testing methods. However, repeatable and reproducible methods for testing product durability under real-life stress conditions are often not available, as in the case of washing machines. The overall objective of this study is to make further progress in the know-how relating to the durability testing of washing machines. A procedure is developed which: 1) consists of washing and spinning cycles, thus imposing thermal and mechanical stresses typically occurring in household washing machines; 2) can be used to monitor the performance of the appliances over time with limited testing burdens. The procedure was applied in a trial with two washing machines for a simulated usage period of two years. Results imply that the procedure can be suitable for evaluating the occurrence of early failures in laboratory testing. The study also provides indications for the further analysis of the repeatability and reproducibility of the test procedure, as well as investigating the consequences associated with longer testing periods. However, a comprehensive assessment of these aspects was not possible in this study. All in all, this guidance could be used by manufacturers and consumer-focussed testing organisations for the assessment and verification of the durability of washing machines, as well as being used to develop standard testing methods for the potential implementation of ecodesign/labelling measures for washing machines.

## Highlights

• Testing methods are needed to support the design of more durable products
• A durability testing procedure for washing machines is developed and applied
• The procedure simulates real life stress conditions over time
• The procedure allow monitoring performance parameters
• The procedure can be used as basis for the development of standard methods

## 1 Introduction

The Communications from the Commission about the Circular Economy Action Plan (European Commission, 2015a) and the Ecodesign Working Plan 2016–2019 (European Commission, 2016) point out the increased importance of improving the resource efficiency of products for promoting a transition towards a more circular economy in the EU. At the macro-level, resource consumption and waste production tend to decrease when product lifetimes increase. Designing more durable products can thus be a key strategy to save materials and to reduce the amount of waste to handle (Alfieri et al., 2018a; Iraldo et al., 2017; Tecchio et al., 2016).

Durability can be defined as the ability to function as required over time under defined conditions of use, maintenance and repair until a limiting state is reached (Alfieri et al., 2018b). The lifetime of a product is a parameter, typically expressed as number of years (or using different units of measure as the number of cycles or the hours of operation), which can serve to orient designers, researchers, policy makers and consumers in their decisions. The lifetime of a product can be influenced by many factors, such as stress, maintenance, technological change, fashion, shift in values and other external environmental influences (Prakash et al., 2020). Depending on the factors considered, a differentiation can be made between technical and functional lifetimes. While the technical lifetime is inherently related to the reliability and resistance of a product until a failure occurs, the functional lifetime is determined by conditions that are created around it (European Environment Agency, 2017). In the following the term durability is used with reference to the technical lifetime. External socio-economic factors that could influence the functional lifetime are not considered (e.g. availability of spare parts and willingness-to-repair a product in cases of failure).

The design of more durable products needs to be supported by sound assessment and verification methods (Dalhammar, 2016; Tecchio et al., 2017a). Internal testing protocols are typically implemented by manufacturers to assess the durability of products and obtain a proxy measure of their technical lifetime. However, there is in general a lack of standardised methods enabling the comparison of different product models on the market. With the implementing decision M/543 (European Commission, 2015b), CEN/CENELEC JTC10 was created at European level to develop horizontal and generic standards on material efficiency aspects for Energy-related Products, including the standard EN 45552:2020 “General method for the assessment of the durability of energy-related products”.

Among energy-related products, household appliances are relatively complex products for which relevant effects on resource consumption and generation of electrical and electronic waste could be minimised through design strategies oriented to ensure the durability of the product (Tecchio et al., 2016, Tecchio et al., 2017b). The importance of durability aspects for washing machines is supported by behavioural research investigating the reasons behind the replacement of different appliances by consumers (Hennies and Stamminger, 2016). It was found that 69% of the interviewed consumers discarded a washing machine because of a defect, thus emphasising the importance of focusing on the technical lifetime of the product. The age of discarded washing-machines varied between 1 and 40 years, with an average of 12 years. However, 50% of discarded washing machines were less than 10 years old, whereas 20% did not reach a lifetime of 5 years. Prakash et al. (2020) carried out an analysis on the disposal of washing machines in Germany. It was found out that about 50% of users had never had their washing machines repaired. Among those who had their washing machines repaired, 13% did it within the warranty period (normally 2 years). Based on such figures it can be roughly estimated that about 6–7% of washing machines may fail during the first 2 years of operation. Lifetime expectations and actual lifetimes were observed to increase with the product purchase price. Consumers seemed in general satisfied with the lifetime of their washing machines when this was longer than 11 years. Such findings are confirmed to a large extent by Seyffert et al. (2018).

The actual lifetime of washing machines has also a clear correlation with the frequency of use (Hennies and Stamminger, 2016), implying that the actual operation of the appliance is the major factor limiting its lifetime.

As summarised by Tecchio et al. (2017b), the most recurring failures of washing machines are related to electronics (e.g. control electronics, control panels, program selectors, relays), shock absorbers, bearings, doors (including seals, handles, hinges and locks) and motors (carbon brushes). An analysis of the failures observed in durability tests conducted on washing machines from 2000 to 2014 in Germany showed that practically all of the main washing machine parts can lead to failure (Prakash et al., 2020).

From an engineering point of view, washing machines are subject to mechanical, thermal and chemical stresses during use. Mechanical and thermal stresses are the main causes of damage (Prakash et al., 2020; Stamminger et al., 2018). Vibrations and other mechanical stresses can impact the whole washing machine (Boyraz and Gunduz, 2013; Tecchio et al., 2017b). Parts that are most exposed to vibrations (tub/drum system, including parts connected to the suds container as motor, transmission belt, springs and shock absorbers) were observed to fail more often than other parts (Prakash et al., 2020). This type of stress occurs on those parts during the washing operation, especially during the spinning phase, due to drum imbalance (Stamminger et al., 2018).

Ways to measure, reduce and control drum imbalance are indispensable for horizontal axis washing machines. Stresses due to the imbalance may be reduced either by decreasing the spinning speed or the duration of spinning at higher speed. However, this could result in an unsatisfactory decrease of the moisture content of the load, which is a key performance element for washing machines (Stamminger et al., 2018). The trade-off between spinning intensity and load moisture content highlights the importance of linking product durability with the fulfilment of performance requirements under real use conditions.

No internationally used standard method is currently available for the assessment of the durability of washing machines (Tecchio et al., 2017b). Consumer testing organisations typically focus on the measurement of washing performance parameters (such as dirt removal, spinning performance, rinse performance, water consumption, energy consumption, ease of use and noise) although they also perform durability tests for washing machines. However, their focus to date has been on component wear-out or failure without considering if the washing performance is negatively affected (Stiftung Warentest, 2013).

An endurance test consisting of 2600 h of continuous cycle operation at 1000 rpm (equivalent to 5200 washing cycles) was run by Ishiyama and Iga (2000), although the tests relevance for real-life operations can be challenged. Park et al. (2006) performed a step-stress test using high temperature and voltage as stress factors in order to verify the operating limit of the pump assembly of a washing machine in a more reasonable amount of time (250–500 h). In an effort to reduce the testing time, accelerated degradation tests (ADT) and accelerated life tests (ALT) were applied by De Carlo et al. (2013) and Tucci et al. (2014), respectively, to estimate the reliability of parts. However, such tests are not relevant for washing machines on the market since an external imbalance control was used. This confirms the difficulty of testing the long-term performance and investigating the failure-time distribution of parts of high-reliability products (Escobar and Meeker, 2007).

Stamminger et al. (2018) worked on a procedure for the accelerated testing of the endurance of washing machines. The procedure consisted of 500 spinning cycles where stresses were applied by a fixed imbalance mass with intermediate washing cycles. This work was important to understand the effects on durability and performance due to mechanical stresses occurring during unbalanced spinning cycles. However, a testing procedure based merely on spinning cycles would induce stresses that are unusual during the use of washing machines.

The aim of this paper is to further develop the know-how relating to the durability testing of washing machines. A procedure is defined to test the durability of washing machines under conditions as far as possible close to real life, as well as based on the monitoring of performance parameters. The procedure is applied to two models of washing machines. Results of the tests are critically discussed and used to draw conclusions and recommendations for possible future improvements and applications (e.g. for the design and assessment of the product or for standardisation and decision making purposes). Indications are also provided for the analysis of the repeatability and reproducibility of the testing procedure, although a comprehensive assessment of these aspects was not possible in this study.

## 2 Methods

Building on the work of Stamminger et al. (2018), an improved durability testing procedure for washing machines was defined and applied to two models on the market (Alfieri et al., 2018b).

Innovative elements were introduced in the development of this new testing procedure to test the entire product under real life conditions, as suggested by Stamminger et al. (2018):

• Coverage of both mechanical and thermal stresses;
• Combination of washing and spinning cycles;
• Avoidance of a fixed imbalance in the spinning cycle.

Moreover, the testing procedure was developed considering the following aspects:

• Monitoring of both failures and loss of performance, as these are possible limiting states due to mechanical and thermal stresses (Stamminger et al., 2018);
• Application of ALT to limit testing costs and reducing the test duration, in order to make the procedure potentially suitable for laboratory testing and verification purposes (Alfieri et al., 2018b);

Additionally, the testing procedure has been kept adherent to the international standards IEC 60546 (IEC, 2010) and EN 60456 (CENELEC, 2016). These are well-established methods to allow repeatable and reproducible results. Adaptations of the requirements set in international standards were however applied (e.g. for the age of the textile load and the hardness of the water used) to allow a time and cost efficient execution of the tests. Such adaptations are unlikely to have a significant influence on the results.

### 2.1 Selection of washing machines

Two models of front loading washing machines were tested. The main characteristics of these washing machines are described in Table 1. Both machines are declared as A+++ energy class with maximum spin speed of 1400 rpm and class B spinning performance.

**Table 1:** Characteristics of the selected washing machines.

Parameter	Device WM-1	Device WM-2
Energy Efficiency class	A+++	A+++
Annual energy consumption	166 kWh	153 kWh
Annual water consumption	9586 L	10560 L
Rated capacity	7 kg	6 kg
Maximum spin speed	1400 rpm	1400 rpm
Spinning performance class	B	B
Price	329 EUR	379 EUR

### 2.2 Durability testing procedure

The testing procedure is structured in the following phases (Alfieri et al., 2018b): (1) Pre-conditioning, set-up and preparation; (2) Initial examination; (3) Testing; (4) Final examination.

#### 2.2.1 Pre-conditioning, set-up and preparation

The machines were equipped with drum speed and temperature measurement devices and installed according to the instructions provided by the manufacturer in the user manual.

The location of the machine was marked on the floor of the laboratory to give an indication of any movement of the machine during the tests.

The test load was conditioned according to EN 60456 (CENELEC, 2016) to have the equilibrium condition (to the defined laboratory climate) for the weight of the load.

#### 2.2.2 Initial examination

An initial visual inspection was carried out in order to verify whether the machines were intact and undamaged, and fit for the testing.

The main washing programmes for cotton load (cotton wash from cold up to 60 °C, separate spinning, separate rinse and spin cycles) were executed with empty load following the instructions from manufacturers. The operation was repeated using any option allowing programme duration reduction. Data was recorded in both sequences.

#### 2.2.3 Testing

The testing consisted in the repetition of two phases:

1. Washing performance tests;
2. Stress tests.

Washing performance tests were executed to check the capability of the washing machine to perform the washing functions before and after the stress induced tests and detect any possible functional losses.

A washing performance test consisted of 10 washing cycles, to be run following closely to the harmonised European measurement standard EN 60456 (CENELEC, 2016), which is suitable for verification purposes. More specifically, the test was composed of:

• 2 cycles at normal cotton 40 °C programme half load;
• 3 cycles at normal cotton 40 °C programme full load;
• 2 cycles at normal cotton 60 °C programme half load; and
• 3 cycles at normal cotton 60 °C programme full load.

Even though time reduction options were available for these programmes, they were not applied in the washing performance tests.

In all wash cycles, test loads and test strips of type 108 (IEC, 2010) from Swissatest Testmaterialien AG batch 120 were applied and evaluated after the test. The following performance parameters were measured for cycles in accordance with EN 60456:

• Total energy consumption (in kWh);
• Total water consumption (in L);
• Cycle duration (in min);
• Water extraction performance (in %);
• Washing performance.

Moreover, other parameters were monitored:

• Actual maximum spin speed (in rpm);
• Duration of maximum spin speed (in s);
• Inlet water temperature (in °C).

Operational data was recorded at 1 s intervals.

The test load was conditioned and normalised following IEC 60456 (IEC, 2010). IEC A∗ detergent was used and dosed according to EN 60456 (CENELEC, 2016). A visual inspection was carried out to detect any leakage.

Stress tests were executed to check the resistance of the machine to typical mechanical and thermal stress conditions occurring during its operation.

The stress tests were 4 blocks of 100 washing and spinning cycles. For each block of cycles, 70% of the cycles were rinse and spin cycles, and the other 30% were washing cycles. The 100 cycles were executed in arbitrary order to allow adaptation to local testing conditions, e.g. to adjust the workflow in an optimal way.

To be as close as possible to real life conditions, the fixed imbalance mass used by Tecchio et al., 2017b was replaced by a textile load to wash and spun at highest possible speed. As the formation of imbalance is known to be higher when the drum is partly filled, a textile mass of two thirds of the rated textile capacity was applied for the rinse and spin cycles.

Washing cycles were cotton programmes run with half load at different temperatures. Cotton programmes are the most used programmes by consumers (Alborzi et al., 2017). They were selected in a way that they were representative for the average washing temperature in Europe of about 40 °C (Alborzi et al., 2017) and close to the average load size of consumers (Kruschwitz et al., 2014).

Programme time reduction options were used for the stress tests in order to reduce the length of the phases which impose less stress (e.g. the washing and rinsing phase), but the maximum spin speed was not reduced.

Table 2 gives an overview of the whole testing procedure. All in all, the testing procedure can be considered representative of approximately 2 years of use of a washing machine (e.g. EU ecodesign and energy labelling requirements for household washing machines assume 220 wash cycles per year (European Commission, 2010a, 2010b)). However, it should be observed that the lifetime target could be varied depending on the availability of resources and on the scope of the analysis (e.g. to verify the occurrence of early failures in worst-performing products on the market or to demonstrate a satisfactorily long lifetime of the product).

**Table 2:** Overview of the durability testing procedure.

Step	Test cycle #	Details of the step
1. Pre-conditioning, set-up and preparation	–	Installation of measurement devices. Marking of the machine on the floor. Conditioning of the load.
2. Initial inspection	–	To be done visually outside and inside the machine. Time-temperature-spin diagrams of all relevant programmes with empty load, with and without applying time reduction options.
3. Testing		
Performance tests (10 cycles) – series I	1–10	Normalisation of the load. Washing cycles: - 2 cycles with normal cotton 40 °C programme at half load, - 3 cycles with normal cotton 40 °C programme at full load, - 2 cycles with normal cotton 60 °C programme at half load, - 3 cycles with normal cotton 60 °C programme at full load.
Stress tests (100 cycles) – series I	11–110	In arbitrary order: 70% rinse and spin cycles (two third load) and 30% washing cycles (half load). Washing cycles: - 10 cycles with normal cotton 40 °C - 10 cycles with normal cotton 30 °C - 5 cycles with normal cotton 60 °C - 5 cycles with normal cotton cold (20 °C) Average temperature of the washing programme = ∼40 °C
Performance tests (10 cycles) – series II	111–120	As for test cycles #1–10.
Stress tests (100 cycles) – series II	121–220	As for test cycles #11–110.
Performance tests (10 cycles) – series III	221–230	As for test cycles #1–10.
Stress tests (100 cycles) – series III	230–330	As for test cycles #11–110.
Performance tests (10 cycles) – series IV	331–340	As for test cycles #1–10.
Stress tests (100 cycles) – series IV	341–440	As for test cycles #11–110.
Performance tests (10 cycles) – series V	441–450	As for test cycles #1–10.
4. Final examination	–	To be done outside and inside of the machine. Parts may be extracted and taken apart.

#### 2.2.4 Final examination

At the end of the test, the following assessment was carried out:

• Analysis of the spin speed profile for all washing and spinning cycles executed during the entire test;
• Analysis of the performance parameters assessed in the five blocks of the washing performance test, each one consisting of 10 washing cycles;
• Analysis of the movement of the machine during the entire set of 450 cycles;
• Analysis of the level of abrasion or wear and tear for all parts which can be visually checked, in comparison with the results of the initial inspection;
• Analysis of the durability of the machine with respect to reference failure and malfunctioning categories (see below).

Reference failures and malfunctioning categories were defined for this analysis based on their severity and ease of recognition:

• Cat. 1: damage to parts of the machine during the test
  - Cat. 1a: damages which cannot be repaired at a reasonable cost (≥50% of the price of the machine);
  - Cat. 1b: damages which can be repaired by a service technician with reasonable cost (<50% of the price of the machine).
• Cat. 2: visible wear and tear of parts of the machine, which neither caused damages nor loss of function, but which may lead to failures at a later stage of use of the machine;
• Cat. 3: misbehaviour of the machine during the tests, like non-conventional noise, vibrations or movements of the machine;
• Cat. 4: loss of functional performance between the initial and the final washing runs. A loss in performance is considered to have occurred if the difference is found to be larger than twice the verification tolerance values as defined in Regulation 1061/2010.

## 3 Results and discussion

### 3.1 Initial examination

The two washing machines underwent an initial visual inspection. There was no visual damage on the packaging or on the machines itself.

All relevant cotton washing programmes were operated once to observe the main characteristics of these programmes. The analysis showed that both machines have different characteristics for how they run cotton programmes and for the sensor systems applied to detect load and, probably, also imbalance. Further details can be found in Alfieri et al. (2018b).

### 3.2 Performance tests

**Fig. 1:**
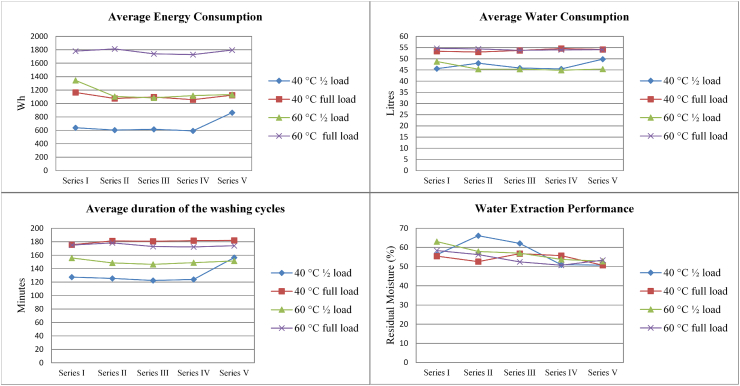
Average behaviour of device WM-1 during the washing performance test cycles in terms of water consumption (upper right), duration of washing cycle (upper left), average energy consumption (lower left) and water extraction (lower right) for the five washing performance test series.

**Fig. 2:**
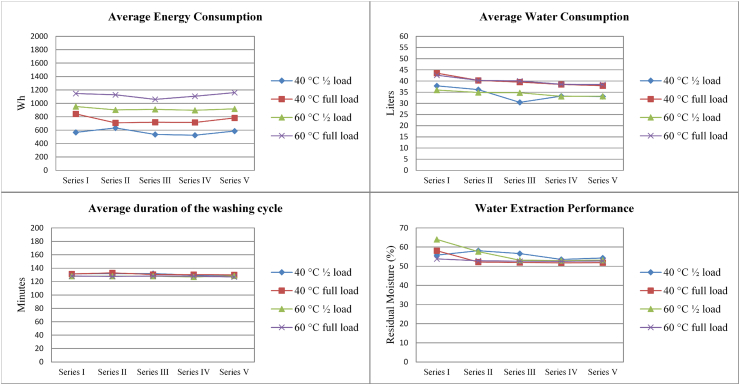
Average behaviour of device WM-2 during the washing performance test cycles in terms of water consumption (upper right), duration of washing cycle (upper left), average energy consumption and (lower left) and water extraction (lower right) for the five washing performance test series.

The results of the performance tests for device WM-1 and device WM-2 are shown in Fig. 1 and Fig. 2. Raw data and parameter variation is expressed as both standard deviation and relative standard deviation (RSD, standard deviation as a % of the average value) in Tables A1–A7 of the Appendix.

Moderate variations in performance are observed in both device WM-1 and device WM-2 for all four parameters across the operated washing cycle series.

Looking at different programmes, the performance parameters generally appear more stable for full loads and with the higher temperature programme (i.e. 60 °C) than half loads with a lower temperature (i.e. 40 °C) for both WM-1 and WM-2. For example, energy consumption RSD was 3% vs. 26% for WM-1 and 4% vs. 10% for WM-2 when comparing the 60 °C full load and 40 °C half load results). Similar trends can be observed for water consumption (RSD = 1% vs. 6% for WM-1 and 4% vs. 11% WM-2) and programme duration (RSD = 2% vs. 16% for WM-1 and 1% vs. 16% WM-2). The higher stability of performance in the case of full loads can be partly attributed to a higher proportion of full-to-half load cycles in the testing (3:2).

In general, WM-1 uses more water and energy than WM-2 for performing a washing cycle. The Sinner’s circle (Sinner, 1960), describing the principles of the washing process, would suggest that reduced uses of water and energy should be compensated by longer cycle times to achieve a given performance. However, this general rule does not apply to WM-1 and WM-2, although the price and characteristics of the two devices can be considered similar (see Table 1).

In terms of water extraction performance, both WM-1 and WM-2 seem to fluctuate moderately over cycles (RSD = 8–12% for WM-1 and 1–5% for WM-2) and to improve progressively (on average) before converging towards a residual moisture content slightly higher than 50%.

The performance parameters described above do not seem affected by the mechanical and thermal stresses applied to WM-1 and WM-2, since no clear performance degradation trends appear from Series I (test cycles 1–10) to Series V (test cycles 441–450).

**Fig. 3:**
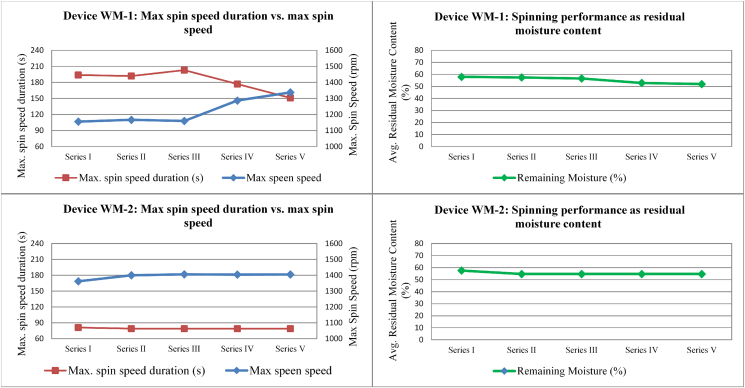
Average “Maximum Spin Speed” versus “Maximum Spin Speed Duration” and resulting average spinning performance of WM-1 and WM-2 for the five washing performance test series.

Results for maximum spin speed and its duration are shown in Fig. 3, and compared with the residual moisture content in order to better understand the behaviour of WM-1 and WM-2.

In the case of WM-1 the claimed spin speed of 1400 rpm was only reached (i.e. with average data exceeding 1300 rpm) in the final series (Series V) of washing performance tests (it was reached in less than 50% of individual cycles in Series I). This is remarkable, as it means that the device WM-1 did not always deliver the claimed spin speed at the beginning of the test. However, the residual moisture content measured did not seem to be as adversely affected by the lower maximum spin speed as might be expected (residual moisture improved from 58% to 52% as average maximum spin speed improved from 1150 to 1350 rpm). This may be explained by the corrective action of the machine itself. Namely, that whenever the maximum spin speed was not reached, the machine automatically prolonged the spinning process and was thus still able to perform a relatively consistent moisture removal. It would be necessary to monitor WM-1 for a longer period to confirm the stability of this behaviour.

On the other hand, device WM-2 always performed in line with claimed performance (in terms of spin speed duration and maximum spin speed) and showed a much more consistant behaviour for these parameters compared to WM-1.

The results show the relevance of the imbalance control procedure for the stress induced to the washing machines. This control procedure influences the distribution of the textile load during the start of the spinning action. When an equal distribution is achieved, the imbalance is low and a high spinning speed may be achieved (allowed). If the load cannot be equally distributed, this imbalance control procedure limits the mechanical stress by modifying the spinning profile, especially by reducing the maximum spin speed. This control procedure allows mechanical stresses to be limited the likelihood of failures reduced (Tecchio et al., 2017b). However, the consumer could experience the problem of not reaching the maximum spin speed if higher moisture contents remain in the load after spinning. Interestingly, one of the devices tested (WM-1) counterbalanced this effect by prolonging the spinning duration when the maximum spin speed could not be reached. On the one hand, this strategy can both guarantee the lifetime of those parts affected by mechanical stress and allow achieving an acceptable spinning performance. On the other hand, the consumer may have to wait longer for the execution of washing programmes.

Coupled with the learning from Stamminger et al. (2018), it has been possible to identify different manufacturer strategies for coping with the effects of imbalance on induced stress and water extraction performance:

• The imbalance does not affect the execution of spinning cycles: declared spinning speeds and water extraction performances are achieved (e.g. WM-2);
• The imbalance affects the execution of the spinning cycles and the machine counter-reacts by reducing the spin speed and prolonging the spinning duration to achieve the desired water extraction performance: although the declared spinning speed is not reached, the declared water extraction performance is achieved (e.g. WM-1).

### 3.3 Stress tests

The maximum spin speeds and spinning duration measured during the tests (Alfieri et al., 2018b) reconfirmed the results achieved in the performance tests: WM-1 did not reach the claimed spinning speed in most of the cycles (probably due to imbalance problems) but tried to compensate the effect of not reaching the maximum speed by prolonging the spinning process at lower spin speed. Device WM-2 reached the maximum spin speed of 1400 rpm in most cycles but stayed at this maximum speed for different times, depending on the programme temperature.

### 3.4 Final examination

For device WM-1, a yellowish colour was observed in the door gasket after the third test run of the initial check of programmes, conducted without load and without detergent. In some tests (nos. 17, 18, 287, 392) the machine moved a few centimetres during the spinning. This was probably due to imbalance conditions which were not detected by the control system of the device. A movement of 10 cm was observed in test no. 186. Interestingly, after test no. 360 (spin cycle) the display did not work properly and the machine did not provide any sound to indicate the end of the cycle. Nevertheless, everything returned to normal conditions after turning the knob.

For device WM-2, abrasions was noted on the door gasket after test no. 3. Further abrasions were visible on the door gasket after test no. 118. This was due to one cloth that was stuck in the door gasket. However, the device was still able to function properly. After test no. 387, white smoke came out of the drum when opening the door. The device was checked by a technician who could not find any defect. No additional malfunctioning was detected in the remaining tests.

Both devices were taken apart for inspection of individual parts. Photographs were taken to compare their state between the initial and the final inspections. A detailed track of visual observations is reported in Alfieri et al. (2018b).

Abrasion was visible in folds of the door gasket of WM-1. Closer inspection revealed that there was a hole in the gasket which could cause a water leakage during washing operation when the water level is sufficiently high (e.g. in synthetic programmes).

Abrasion was visible also in folds of the door gasket of device WM-2. This was likely to be due to clothes stuck in between the gasket and the drum. To try to identify the origin of the smoke observed in test no. 387, the tub of WM-2 was dismounted and opened. No indication of any failure mechanism could be found. However, at the outer side of the front drum ring, a thin grey layer was observed and wiped away.

Some additional abrasion was observed on the heating elements of both WM-1 and WM-2 but they were not considered to affect the technical lifetime of the machines.

To sum up, the following failures/malfunctioning categories were detected in the two devices:

• WM-1 had a category 2 failure (a hole in the door gasket which could lead to a water leakage during the lifetime of the appliance) and a category 3 event (occasional movement during the operation).
• WM-2 had two category 1b failures (a cloth stuck in the door gasket that was worn out and soiled by rubber abrasion; a smell that appeared after opening the door).

No loss of function was observed for either WM-1 or WM-2 at the end of the tests (category 4 failure) and no significant failure of electronic parts was observed, at least for the representative time of this experiment (∼2 years).

### 3.5 Repeatability and reproducibility of the testing procedure

The testing procedure was designed to stress washing machines in conditions that are very close to real life use, and to allow the measurement of their performance. This was based on the IEC 60456 and EN 60456 standards, which describe how to test washing machines and deliver results that are repeatable (i.e. almost the same results are obtained when the test is repeated later in time in the same laboratory) and reproducible (i.e. almost the same results are obtained when the test is repeated in another laboratory) (IEC, 2015). Modifications were introduced for speeding up the testing procedure (i.e. use of normal programmes in stress tests) and reducing the testing costs (i.e. use of normal textiles for stress test). Although testing two single devices cannot demonstrate the repeatability and reproducibility of results (which is out of the scope of this work), it can be at least considered that a testing procedure built-up on standards used internationally is expected to lead to repeatable and reproducible results.

## 4 Conclusions

The purpose of this work was to develop further knowledge about how to assess and verify the durability of washing machines, expressed in terms of technical lifetime.

A new testing procedure was developed and applied to two washing machines. The experience gained allows highlighting the importance of linking durability tests to real life conditions and performance of the product. The main strengths of the procedure developed in this work are that:

• It is potentially suitable for laboratory testing since it applies accelerated life testing for reducing test duration and costs as far as reasonable;
• It simulates thermal and mechanical stresses occurring in household washing machines in real life conditions,
• It includes the monitoring of washing performance over time.

Such aspects are important since they allow taking into consideration how the imbalance control systems are able to handle the stress induced during the operation of washing machines and how the performance is affected.

As defined, the procedure covers the first 2 years of use of a washing machine (the average lifetime of the product is about 12 years) and could be useful for evaluating the occurrence of early failures in a relatively limited amount of time. Based on the evidence available, the authors consider that statistics about early failures may differ significantly, depending on the quality of devices. However, manufacturers keep this information confidential for sensitivity reasons.

This study as such did not assess if results are repeatable and reproducible, and do not analyse the behaviour of the tested devices for longer periods of use. However, the testing procedure built-up on standards used internationally to produce repeatable and reproducible results for the performance of washing machines. A broader analysis would be needed for concluding about the repeatability and reproducibility of the testing procedure. At least three samples of the same washing machine model should be tested in parallel, and in different laboratories.

The procedure could potentially cover more extended periods of use (e.g. to verify a satisfactorily-long technical lifetime of the product). It is worth noting that the testing procedure described in this work has already required a considerable amount of time (in total 697 h for one person running two devices in parallel). This serves to highlight the importance of human resource availability and the need for cooperation and knowledge sharing in the development of widely agreed methods (e.g. with manufacturers and testing organisations).

Considering that testing the durability of washing machines is a lengthy process, options to reduce the testing time could include:

• Leave out or reduce the number/frequency of performance tests in between stress tests;
• Use shorter programme options for the performance tests;
• Leave out the intermediate (spin) drying of the load between different stress test cycles.

If points 1 and 3 are implemented, robots could be potentially used for restarting programmes during the stress tests (rinse and spin cycles, and washing cycles), with possible saving of costs and time since only the execution of the washing performance tests at the start and at the end of the stress tests would necessarily need an operator. It is estimated that the overall duration of the test could potentially decrease by one third.

Future research efforts should be oriented towards the analysis of the repeatability and reproducibility of the testing procedure, and its application to a larger sample of devices over longer periods of time. The analysis of the functional performance of washing machines after the first 2 years of use, as well as the potential occurrence of major failures, can allow understanding which parameters to monitor, and for how long, in the procedure.

Finally, it is recommended to align the performance testing methods with those used for regulatory purposes in a certain market (e.g. those resulting from the final revision of the EU ecodesign and energy labelling requirements for household washing machines).

All in all, this work can act as a guide to manufacturers and consumer-focussed testing organisations for the assessment of the durability of washing machines. Moreover, if integrated in a standardised method, these could support the potential implementation of regulatory measures contributing to the prevention of premature obsolescence of washing machines. This can be of particular importance for a product group such as washing machines, in which obsolescence is mainly associated to technical aspects, while a less relevant role is played by other factors (e.g. technological change, fashion).

## CRediT authorship contribution statement

**Rainer Stamminger:** Methodology, Resources, Validation, Writing - original draft. **Alexander Bues:** Investigation, Data curation. **Felice Alfieri:** Conceptualization, Supervision, Formal analysis, Writing - original draft. **Mauro Cordella:** Conceptualization, Supervision, Project administration, Writing - original draft.

## Declaration of competing interest

The authors declare that they have no known competing financial interests or personal relationships that could have appeared to influence the work reported in this paper.

## Figures and Tables

**Table A.1:** Energy consumption values measured for each washing cycle for the washing performance test - WM-1 and WM-2

Energy (Wh)	40 °C ½ load	40 °C ½ load	40 °C 1 load	40 °C 1 load	40 °C 1 load	60 °C ½ load	60 °C ½ load	60 °C 1 load	60 °C 1 load	60 °C 1 load	Avg. of 10 runs
Device WM-1											
1–10	647	631	1083	1202	1206	1643	1047	1683	1814	1844	1280
111–120	599	608	1084	1035	1107	1079	1130	1813	1804	1825	1208
221–230	605	627	1119	1110	1062	1112	1059	1773	1729	1719	1191
331–340	602	582	1091	1017	1064	1137	1099	1787	1712	1689	1178
441–450	1148	581	1090	1119	1165	1155	1112	1793	1797	1799	1276
Avg.	663		1103			1157		1772			
Std.	172		54			174		52			
Std./Avg. (%)	26%		5%			15%		3%			
Device WM-2											
1–10	536	600	845	852	832	946	962	1200	1239	1275	929
111–120	652	614	625	757	747	915	890	1134	1212	1290	883
221–230	470	604	679	741	737	894	927	1246	1156	1160	861
331–340	568	482	767	684	692	927	867	1192	1229	1227	863
441–450	588	584	748	795	802	938	900	1258	1268	1295	918
Avg.	570		754			917		1225			
Std.	58		65			29		50			
Std./Avg. (%)	10%		9%			3%		4%			

**Table A.2:** Water consumption values measured for each washing cycle for the washing performance test - WM-1 and WM-2

Water (L)	40 °C ½ load	40 °C ½ load	40 °C 1 load	40 °C 1 load	40 °C 1 load	60 °C ½ load	60 °C ½ load	60 °C 1 load	60 °C 1 load	60 °C 1 load	Avg. of 10 runs
Device WM-1											
1–10	45.7	45.5	52.6	53.1	54.5	53.0	44.5	55.7	54.5	53.7	51.3
111–120	46.4	49.6	54.7	49.7	54.6	45.5	45.2	54.2	53.3	55.7	50.9
221–230	45.3	46.4	53.2	53.9	54.1	46.0	44.7	53.5	54.4	53.4	50.5
331–340	44.8	46.1	54.4	54.6	54.7	45.3	44.5	53.7	53.7	54.6	50.6
441–450	54.3	45.4	54.6	53.8	53.9	45.3	45.5	53.3	53.7	55.2	51.5
Avg.	47.0		53.8			46.0		54.2			
Std.	2.9		1.3			2.5		0.8			
Std./Avg. (%)	6%		2%			5%		1%			
Device WM-2											
1–10	37.6	38.1	43.5	44.1	43.0	35.7	36.3	41.2	43.2	43.4	40.6
111–120	36.0	36.3	39.5	40.3	41.1	34.6	35.2	40.3	39.8	40.7	38.4
221–230	24.9	36.0	39.3	39.6	39.7	34.4	35.1	41.0	39.4	39.8	36.9
331–340	33.9	32.6	39.1	38.7	37.6	33.1	33.2	38.5	38.6	38.6	36.4
441–450	33.0	33.2	36.4	38.5	38.8	33.2	33.2	38.4	38.3	38.6	36.2
Avg.	34.2		40.0			34.4		40.0			
Std.	3.8		2.2			1.2		1.7			
Std./Avg. (%)	11%		5%			3%		4%			

**Table A.3:** Duration of each washing cycle for the washing performance test - WM-1 and WM-2

Duration (min)	40 °C ½ load	40 °C ½ load	40 °C 1 load	40 °C 1 load	40 °C 1 load	60 °C ½ load	60 °C ½ load	60 °C 1 load	60 °C 1 load	60 °C 1 load	Avg. of 10 runs
Device WM-1											
1–10	130	125	128	199	200	166	146	168	178	179	162
111–120	126	125	191	199	199	146	151	178	180	177	167
221–230	120	125	202	203	191	148	145	173	174	172	165
331–340	123	125	198	197	200	148	150	174	172	171	166
441–450	192	121	194	196	196	154	149	173	175	174	172
Avg.	131		193			150		175			
Std.	22		18			6		3			
Std./Avg. (%)	16%		9%			4%		2%			
Device WM-2											
1–10	130	133	131	130	133	127	129	128	129	128	130
111–120	131	133	130	132	137	128	128	127	129	127	130
221–230	132	132	131	131	130	128	128	129	128	128	130
331–340	130	129	129	131	131	126	128	129	127	128	129
441–450	130	130	129	131	130	128	129	128	126	127	129
Avg.	131		131			128		128			
Std.	1		2			1		1			
Std./Avg. (%)	1%		2%			1%		1%			

**Table A.4:** Maximum spin speed measured in each washing cycle for the washing performance test - WM-1 and WM-2

Max. spin speed (rpm)	40 °C ½ load	40 °C ½ load	40 °C 1 load	40 °C 1 load	40 °C 1 load	60 °C ½ load	60 °C ½ load	60 °C 1 load	60 °C 1 load	60 °C 1 load	Avg. of 10 runs
Device WM-1											
1–10	1382	1030	1032	1334	1372	1020	1020	1020	969	1381	1156
111–120	870	962	1247	1371	1322	1385	994	1295	914	1310	1167
221–230	920	960	1010	992	1386	995	1389	1386	1243	1315	1160
331–340	1385	1384	984	1010	1388	1198	1388	1362	1386	1381	1287
441–450	1393	1388	1392	1386	1355	1346	1390	1353	1344	1028	1338
Avg.	1167		1239			1213		1246			
Std.	234		175			186		170			
Std./Avg. (%)	20%		14%			15%		14%			
Device WM-2											
1–10	1401	1400	1401	1400	1403	995	1405	1404	1404	1403	1362
111–120	1405	1401	1406	1407	1404	1403	1405	1364	1403	1402	1400
221–230	1407	1405	1409	1405	1409	1408	1403	1406	1405	1407	1406
331–340	1405	1406	1407	1406	1404	1402	1404	1404	1403	1403	1404
441–450	1408	1409	1406	1409	1409	1407	1404	1375	1408	1411	1405
Avg.	1405		1406			1364		1400			
Std.	3		3			130		13			
Std./Avg. (%)	0%		0%			9%		1%			

**Table A.5:** Duration of max. spin speed in each washing cycle for the washing performance test - WM-1 and WM-2

Duration of max. spin speed (s)	40 °C ½ load	40 °C ½ load	40 °C 1 load	40 °C 1 load	40 °C 1 load	60 °C ½ load	60 °C ½ load	60 °C 1 load	60 °C 1 load	60 °C 1 load	Avg. of 10 runs
Device WM-1											
1–10	142	273	172	142	135	272	264	134	267	136	194
111–120	287	168	242	131	140	138	273	146	258	139	192
221–230	146	278	269	253	139	270	141	139	256	143	203
331–340	141	139	267	268	139	265	141	134	138	139	177
441–450	142	143	136	136	134	145	142	131	138	258	151
Avg.	186		180			205		170			
Std.	65		59			67		56			
Std./Avg (%)	35%		33%			33%		33%			
Device WM-2											
1–10	93	92	92	92	93	74	66	67	68	68	81
111–120	91	92	91	91	92	66	66	70	66	68	79
221–230	91	92	93	91	93	66	66	66	69	66	79
331–340	92	92	92	91	92	67	67	68	65	66	79
441–450	93	92	92	91	91	67	66	67	66	67	79
Avg.	92		92			67		67			
Std.	1		1			2		1			
Std./Avg (%)	1%		1%			4%		2%			

**Table A.6:** Water extraction performance at the end of each washing cycle for the washing performance test – WM-1 and WM-2

Water extraction performance (%)	40 °C ½ load	40 °C ½ load	40 °C 1 load	40 °C 1 load	40 °C 1 load	60 °C ½ load	60 °C ½ load	60 °C 1 load	60 °C 1 load	60 °C 1 load	Avg. of 10 runs
Device WM-1											
1–10	53.9	58.5	59.9	53.0	53.6	65.3	60.8	62.2	60.4	52.6	58.0
111–120	70.5	61.8	53.8	50.1	54.0	55.4	60.5	53.1	63.0	52.8	57.5
221–230	65.7	58.6	59.1	60.0	51.3	52.3	61.8	50.0	52.9	54.6	56.6
331–340	51.7	50.3	58.7	58.2	50.4	56.9	50.7	51.2	50.3	50.7	52.9
441–450	52.6	49.0	50.0	50.5	51.7	55.1	50.6	51.5	51.7	57.0	52.0
Avg.	57.3		54.3			56.9		54.3			
Std.	7.1		3.8			5.0		4.3			
Std./Avg (%)	12%		7%			9%		8%			
Device WM-2											
1–10	54.2	57.5	66.8	53.9	53.7	67.3	60.8	53.2	54.4	54.0	57.6
111–120	53.5	59.1	52.9	52.1	53.1	54.4	61.2	54.3	53.1	53.3	54.7
221–230	57.1	56.2	51.4	52.2	52.3	59.7	54.1	52.3	52.3	53.3	54.1
331–340	56.5	50.6	51.5	51.8	52.1	59.0	53.7	52.2	52.6	52.9	53.3
441–450	56.9	51.8	50.9	52.1	52.7	59.9	53.3	53.1	52.5	52.7	53.6
Avg.	55.3		53.3			58.3		53.1			
Std.	2.7		3.8			4.5		0.7			
Std./Avg (%)	5%		7%			8%		1%			

**Table A.7:** Washing performance in each washing cycle for the washing performance test – WM-1 and WM-2

Washing performance	40 °C ½ load	40 °C ½ load	40 °C 1 load	40 °C 1 load	40 °C 1 load	60 °C ½ load	60 °C ½ load	60 °C 1 load	60 °C 1 load	60 °C 1 load	Avg. of 10 runs
Device WM-1											
1–10	0.98	0.99	1.01	1.02	1.03	1.10	1.07	1.05	1.06	1.06	1.04
111–120	1.03	1.03	1.03	1.03	1.04	1.14	1.12	1.07	1.07	1.07	1.06
221–230	0.97	0.99	1.02	1.02	1.03	1.13	1.09	1.05	1.07	1.07	1.04
331–340	1.00	1.01	1.01	1.01	1.02	1.11	1.12	1.05	1.06	1.07	1.05
441–450	1.08	1.00	1.01	1.02	1.02	1.12	1.13	1.05	1.06	1.06	1.06
Avg.	1.01		1.02			1.11		1.06			
Std.	0.03		0.01			0.02		0.01			
Std./Avg (%)	3%		1%			2%		1%			
Device WM-2											
1–10	1.02	1.02	0.98	0.98	0.99	1.08	1.08	1.00	1.03	1.02	1.02
111–120	1.01	1.03	0.97	0.96	0.97	1.08	1.10	1.01	1.02	1.05	1.02
221–230	1.02	1.03	0.96	0.98	0.99	1.08	1.12	1.02	1.03	1.04	1.03
331–340	1.00	1.01	0.97	0.97	0.97	1.11	1.09	0.99	1.01	1.04	1.01
441–450	1.01	1.01	0.96	0.99	0.99	1.10	1.09	1.03	1.04	1.04	1.03
Avg.	1.02		0.97			1.09		1.02			
Std.	0.01		0.01			0.01		0.02			
Std./Avg (%)	1%		1%			1%		2%			
